# Correlation of diabetes and adverse outcomes in hospitalized COVID-19 patients admitted to a tertiary hospital in China during a small-scale COVID-19 outbreak

**DOI:** 10.7717/peerj.18865

**Published:** 2025-01-27

**Authors:** Yu Li, Guanni Li, Jiahong Li, Zirui Luo, Yaxuan Lin, Ning Lan, Xiaodan Zhang

**Affiliations:** 1 Department of Endocrinology, The Second Affiliated Hospital, Guangzhou Medical University, Guangzhou, China; 2 The Second Clinical Medicine School, Guangzhou Medical University, Guangzhou, China

**Keywords:** Diabetes, COVID-19, Diabetic complications, Mortality, Risk factors

## Abstract

**Background:**

The aim of this study was to investigate the impact of diabetes on mortality and adverse outcomes in COVID-19 patients and to analyse the associated risk factors.

**Methods:**

This is a retrospective cohort study in 500 hospitalized patients with COVID-19 infection (214 with diabetes and 286 without diabetes) admitted to a tertiary hospital in China from December 2022 to February 2023. Demographic information, clinical characteristics and outcomes were collected. Survival status was investigated at discharge and at 6 months after discharge.

**Results:**

The mortality rate of COVID-19 patients with diabetes was higher than the rate of non-diabetic COVID-19 patients, both at discharge, and at 6 months after discharge. Body mass index (BMI), C-reactive protein (CRP), pH, D-dimer, blood osmotic pressure, serum creatinine, white blood cell count, creatine kinase and hospitalization expenses were significantly different between diabetic group and non-diabetic group (*p* < 0.05). Compared with the survivors, non-survived COVID-19 patients with diabetes had worse diabetes control indicators, with random blood glucose increased by 3.58 mmol/L (*p* < 0.05), and fasting blood glucose increased by 2.77 mmol/L (*p* < 0.01). In addition, there were significant differences in age, heart rate, CRP, pH, potassium (K^+^), serum creatinine, white blood cell count, creatine kinase, the proportion with diabetic complications, treatment in ICU and mechanical ventilation between survivors and non-survivors of COVID-19 patients with diabetes. By multivariate logistic regression analysis, the death of COVID-19 patients with diabetes is positively correlated with age and CRP (*p* < 0.05), and has a trend towards significance with fasting blood glucose (*p* < 0.1).

**Conclusion:**

Infection with COVID-19 on the basis of diabetes can significantly increase mortality, which was further associated with diabetes control indicators.

## Introduction

The coronavirus pneumonia broke out in 2019 was given rise by severe acute respiratory syndrome coronavirus 2 (COVID-19) which is a new enveloped RNA beta-coronavirus (Zhou et al., 2021; Shi et al., 2024). The rapid spread and significant harm of COVID-19 have made it a public health emergency of great international concern in recent years. The World Health Organization (WHO) declared COVID-19 an epidemic on March 11, 2020 (Mohammadian Erdi et al., 2023). According to incomplete statistics of WHO, until April 2023, there have been more than 760 million confirmed cases of COVID-19 and 6.8 million deaths worldwide. The outbreak of COVID-19 has caused havoc to the global social and health care system (Krett, Salter & Newsome, 2024).

Diabetes mellitus is a complex clinical syndrome, which is characterized by the disorder of glucose metabolism caused by the interaction of genetic and environmental factors (Hou et al., 2023). In view of the worldwide increasing incidence of diabetes, which has brought great challenges to public health, diabetes has become a health problem of global concern. The disorder of glucose and lipid metabolism caused by insulin resistance and β-cell dysfunction exert harmful effects on host immunity, including functional defects of immune cells, abnormal production of inflammatory cytokines, and imbalance of immune response, which increase the incidence of various infections, post-infection complications, and even death resulting from serious infection (Zhou et al., 2021, 2024). In COVID-19 outbreaks, studies have shown that about half of the patients hospitalized with coronavirus pneumonia had co-morbidities, and diabetes is one of the major co-morbidities. The mortality associated with COVID-19 was much higher in patients with diabetes than that in nondiabetic population (Chen et al., 2020; Huang et al., 2020; Yang et al., 2020).

Due to the effective prevention and quarantine measures of the government, COVID-19 was not widespread in communities in China before 2022, with a quite low infection rate. Nevertheless, along with the updated epidemic prevention policy in China in December 2022, China experienced a nationwide epidemic outbreak of COVID-19, lasting from December 2022 to January 2023, at a time when Omicron was the predominant circulating variant (Joint Prevention and Control Mechanism of the State Council, 2022; World Health Organization, 2023). We observed that nearly half of the infected hospitalized patients have diabetes. Although the characteristics, risk factors and adverse outcomes associated with COVID-19 have been widely reported, studies in Chinese population are relatively rare compared with the ones in other regions, especially relative to our large population. In addition, the early studies regarding COVID-19 were published mainly at the beginning of the outbreak during 2019–2020, in a time with limited precautions and understanding about the disease. As time passes by, the public have received widespread vaccination against COVID-19 and accumulated rich experience in COVID-19 prevention (Chinese Center for Disease Control and Prevention, 2023). However, when consecutively facing real patients with COVID-19 infections coming from communities and with complex comorbities, we feel a lack of experience in management. Our hospital is a large tertiary hospital in South China with over ten thousand discharges per year. During the epidemic outbreak, we received a relatively large number of COVID-19 patients, most of whom were the first time infected and experienced obvious symptoms. With regard to the possible disparities regarding COVID-19 infections among different areas and populations, we considered it necessary to summarize the characteristics and outcomes of COVID-19 patients admitted to our hospital during this special epidemic outbreak, to investigate the impact of diabetes on mortality and adverse outcomes in Chinese COVID-19 patients in South China and to analyse the associated risk factors.

## Materials and Methods

### Study population

We conducted a retrospective cohort study of hospitalized patients with confirmed COVID-19 infection admitted to the Second Affiliated Hospital, Guangzhou Medical University, between December 2022 and February 2023. The study was approved by the Ethics Committee of the Second Affiliated Hospital, Guangzhou Medical University (Approval Number 2023-hg-ks-51). Since the study is retrospective and the data are anonymous, written informed consent was not required. The study complied with the requirements of Helsinki Declaration of the World Medical Association. Inclusion criteria were adults with COVID-19 infection confirmed by real-time reverse transcriptase PCR testing. Patients who have been previously diagnosed with diabetes and/or subjects with HbA_1c_ ≥ 6.5% were considered to have diabetes (American Diabetes Association, 2018). Exclusion criteria were as follows: (1) individuals in asymptomatic infection state; (2) pregnant women; (3) patients with age < 18 years. Finally, 500 patients were consecutively enrolled for this study.

### Data collection

Electronic health records were reviewed, including clinical electronic medical records, nursing records, laboratory examination results and treatment records. We collected age, gender, body mass index (BMI), diabetic complications (including diabetic ketoacidosis, hyperglycemic hyperosmolar status, diabetic kidney disease, diabetic retinopathy and diabetic peripheral neuropathy), length of hospital stay, hospitalization expenses, duration of symptoms prior to admission, vital signs (heart rate, blood pressure), laboratory examinations at the initial stage of admission (HbA_1c_, fasting blood glucose, random blood glucose at admission, C-reactive protein (CRP), white blood cell (WBC) count, neutrophil count, lymphocyte count, platelet count, pH, HCO_3_^−^, anion gap, blood osmotic pressure, blood sodium, blood potassium, serum creatinine, D-dimer, procalcitonin, troponin, NT-proBNP, creatine kinase (CK), creatine kinase-myocardial band (CK-MB)) and specific treatments (mechanical ventilation, treatment in ICU and use of steroids). Survival status was determined at discharge and at 6 months after discharge by telephone follow-up.

### Statistical analysis

Continuous variables are presented by median and interquartile range (IQR) under non-normal distribution, and by mean and standard deviation (SD) under normal distribution. Chi-squared test was used for comparison of categorical variables and to calculate odds ratio (OR) for predicting death. Nonparametric paired Mann Whitney U-test and Student’s t test were used for comparison of continuous data. To further identify factors associated with death, multivariate logistic regression analysis was performed with the variable (age, heart rate, fasting blood glucose, CRP, serum creatinine, D-dimer, anion gap, platelet count, CK-MB) with *p* < 0.05 in the independent variable in univariate analysis after empirical exclusion of collinearity. And logistic regression analysis was used to investigate mortality differences between those with and without diabetes. The Benjamini-Hochberg correction was used for multiple comparison adjustments. The significance level was set at adjusted *p*-value < 0.05, and a trend towards significance was considered at adjusted *p*-value < 0.1. The receiver operating characteristic (ROC) analysis was conducted to analyze the prediction accuracy of risk factors (*p* < 0.1) in the multivariate logistic regression analysis and to calculate cut-off values. The Kaplan-Meier survival curves were constructed according to the cut-off values to evaluate the influence of different risk factors (age, CRP, fasting blood glucose) on the survival of patients. SPSS software (version 26) was used for statistical analysis. GraphPad Prism 8.0.2 was used for graph production.

## Results

### Characteristics and outcomes of COVID-19 survivors and non-survivors

**Figure 1:**
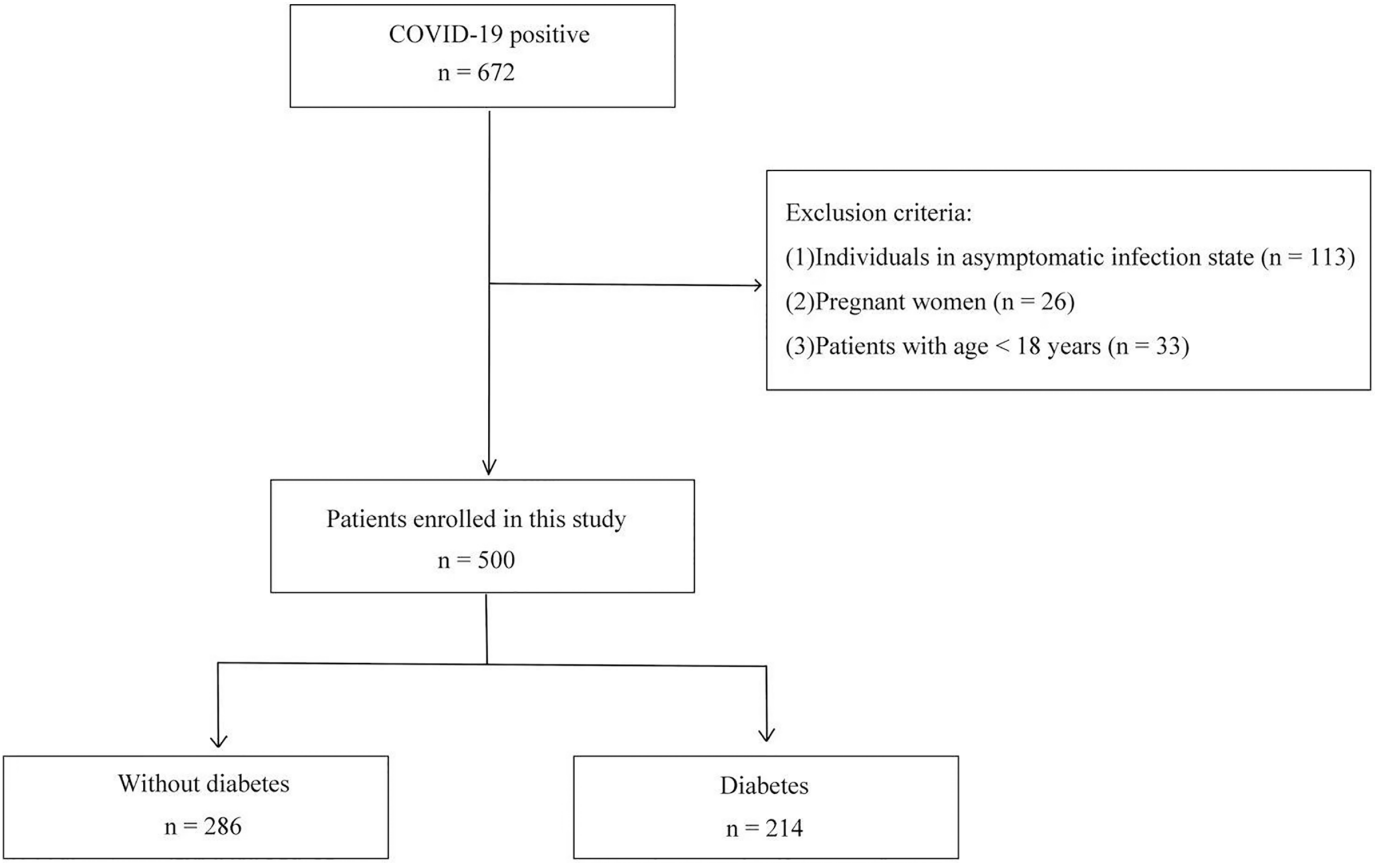
Flowchart of subject inclusion.

A total of 500 patients with COVID-19 infection (214 with diabetes and 286 without diabetes) were included in the study. Figure 1 presents the flowchart of subject selection. Among the COVID-19 patients, the median age was 70 years old, ranging from 23 to 100 years old. Males constituted the majority (*n* = 349, 69.8%). The mortality was 22% (390 survivors *vs*. 110 non-survivors). The median age of non-survivors was 83 years, 13 years higher than that of survivors (*p* < 0.001). The sex distribution, BMI, blood pressure, pH, platelet count, troponin and length of hospital stay were comparable between survivors and non-survivors. Nevertheless, duration of symptoms before admission, heart rate, HbA_1c_, random blood glucose, fasting blood glucose, CRP, HCO_3_^−^, anion gap, blood osmotic pressure, blood sodium, blood potassium, serum creatinine, D-dimer, procalcitonin, WBC count, neutrophil count, lymphocyte count, NT-proBNP, CK, CK-MB and hospitalization expenses were significantly different between survivors and non-survivors (*p* < 0.05). Detailed information for characteristics and outcomes of COVID-19 survivors and non-survivors was shown in Table 1.

**Table 1:** Characteristics and outcomes of COVID-19 survivors and non-survivors.

Characteristics	Total (*n* = 500)	Survivor (*n* = 390)	Non-survivor (*n* = 110)	*p*-value
Demographic characteristics				
Age (year)	74 (60, 84)	70 (58, 82)	83 (74, 83)	0.000
Gender				0.774
Male (*n*)	349 (69.8%)	271 (77.7%)	78 (22.3%)	
Female (*n*)	151 (30.2%)	119 (78.8%)	32 (21.2%)	
BMI (kg/m^2^)	23.03 (20.26, 25.32)	23.03 (20.33, 25.34)	23.08 (19.16, 27.00)	0.970
Clinical characteristics				
Duration of symptoms prior to admission (day)	7 (5, 13)	8 (5, 14)	7 (4, 10)	0.010
Diabetes (n)	214 (42.8%)	155 (72.4%)	59 (27.6%)	0.009
Systolic blood pressure (mmHg)	131 (120, 144)	131 (120, 143)	135 (113, 156)	0.132
Diastolic blood pressure (mmHg)	78 (70, 86)	78 (71, 9)	79 (66, 91)	0.970
Heart rate (beat/min)	85 (77, 97)	84 (77, 95)	93 (75, 111)	0.000
HbA_1c_ (%)	6.60 (6.00, 7.50)	6.50 (5.90, 7.38)	6.90 (6.20, 8.95)	0.013
Random plasma glucose (mmol/L)	7.79 (6.10, 11.27)	7.60 (6.02, 10.69)	8.95 (6.70, 14.67)	0.002
Fasting plasma glucose (mmol/L)	6.27 (5.11, 8.73)	5.97 (5.01, 8.27)	7.69 (5.58, 12.00)	0.000
CRP (mg/L)	54.7 (12.7, 129.7)	42.0 (10.3, 108.9)	111.5 (27.7, 195.4)	0.000
pH	7.41 (7.36, 7.45)	7.41 (7.37, 7.45)	7.39 (7.34, 7.44)	0.106
HCO_3_^−^ (mmol/L)	23.30 (19.20, 25.50)	23.60 (19.90, 25.78)	21.00 (15.73, 26.27)	0.000
Anion gap (mmol/L)	11.20 (9.40, 13.60)	10.90 (9.30, 12.65)	12.80 (10.48, 15.55)	0.000
Osmotic pressure (/kg(water))	282 (276, 292)	280 (275, 286)	295 (281, 314)	0.000
Na^+^ (mmol/L)	138 (135, 141)	137.7 (134.5, 140.8)	139.0 (136.3, 143.8)	0.000
K^+^ (mmol/L)	3.87 (3.52, 4.33)	3.82 (3.51, 4.23)	4.05 (3.63, 4.65)	0.000
Serum creatinine (μmoI/L)	91 (70, 151)	84 (67, 124)	151 (90, 243)	0.000
D-dimer (mg/L)	0.94 (0.45, 2.04)	0.73 (0.41, 1.49)	2.11 (1.04, 8.31)	0.000
Procalcitonin (ng/ml)	0.13 (0.06, 0.50)	0.10 (0.05, 0.29)	0.57 (0.16, 2.74)	0.000
White blood cell count (10^9^/L)	7.33 (4.97, 10.16)	6.89 (4.65, 9.24)	9.56 (6.57, 13.23)	0.000
Neutrophil (10^9^/L)	5.56 (3.40, 8.59)	5.08 (3.10, 7.64)	7.77 (5.18, 11.51)	0.000
Lymphocyte (10^9^/L)	0.82 (0.53, 1.26)	0.90 (0.57, 1.35)	0.73 (0.45, 0.97)	0.000
Platelet count (10^9^/L)	202 (152, 272)	203 (155, 274)	200 (148, 270)	0.826
Troponin (μg/L)	0.07 (0.01, 7.15)	0.06 (0.01, 8.325)	0.09 (0.04, 0.93)	0.259
NT-proBNP (ng/L)	164 (50, 795)	100 (38, 463)	738 (178, 2,555)	0.000
Creatine kinase (U/L)	94 (52, 227)	79 (49, 183)	181 (70, 379)	0.000
Creatine phosphokinase isoenzyme (U/L)	14 (10, 19)	13 (10, 18)	16 (12, 26)	0.000
Length of stay in hospital (day)	9 (7, 15)	10 (7, 15)	9 (4, 14)	0.066
Hospitalization expenses ($)	2,087 (1,312, 3,603)	1,976 (1,304, 3,404)	2,516 (1,434, 4,389)	0.025
Therapy				
Treatment in ICU (*n*)	27 (5.4%)	9 (33.3%)	18 (66.7%)	0.000
Use of steriods (*n*)	251 (50.2%)	187 (74.5%)	64 (25.5%)	0.058
Mechanical ventilation (*n*)	48 (9.6%)	23 (47.9%)	25 (52.1%)	0.000

**Note:**

Data were presented as *n* (%) or mean ± SD or median (range) unless otherwise stated. Abbreviations: BMI, body mass index; CRP, C-reactive protein.

**Figure 2:**
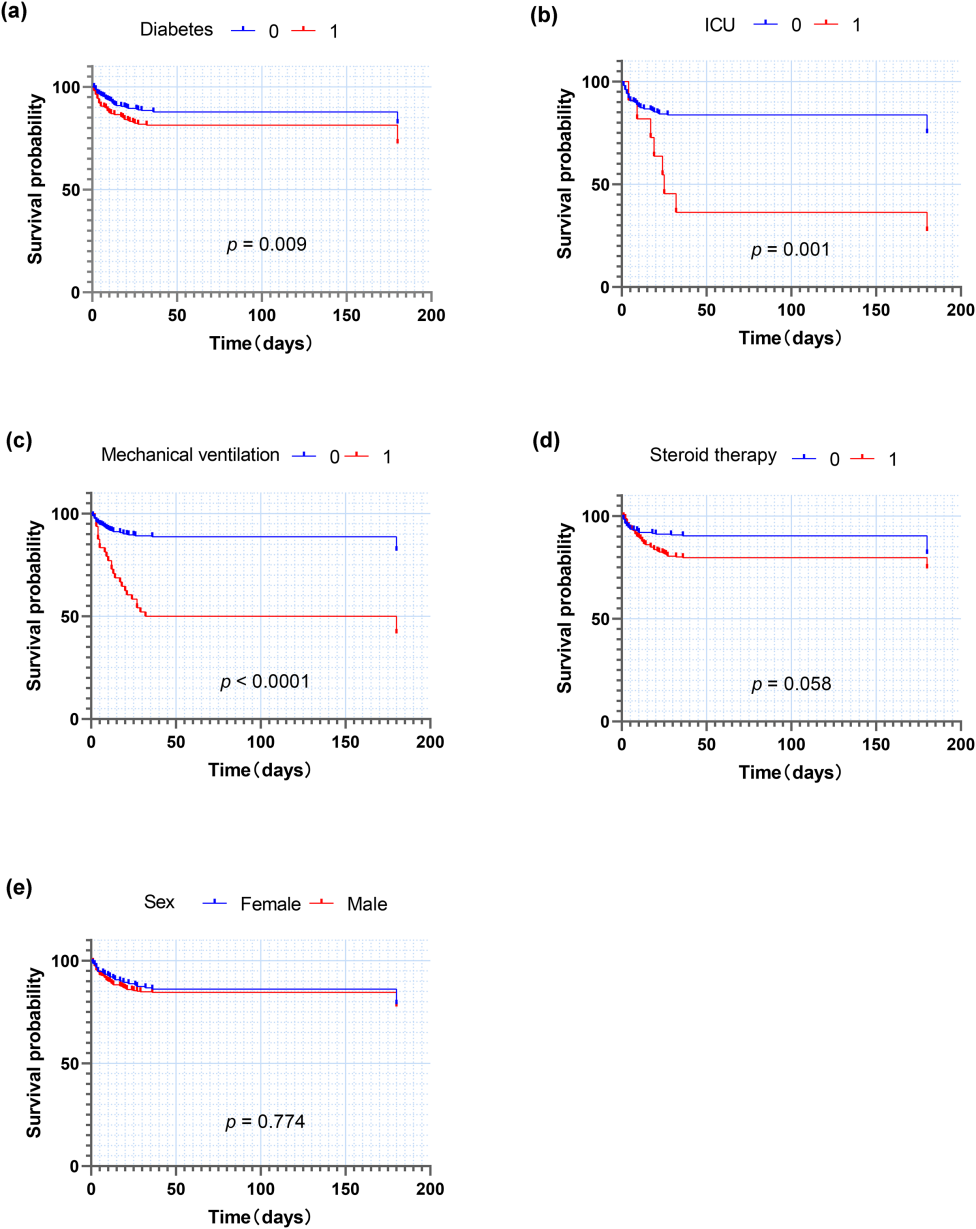
Kaplan-Meier analysis for death of patients after COVID-19 infection. (A) Diabetes; (B) ICU; (C) Mechanical ventilation; (D) Steriods therapy; (E) Age (year). Abbreviations: ICU, Treatment in ICU.

In addition, we divided the patients into groups according to whether they had diabetes, whether they received treatment in ICU or mechanical ventilation or steroids therapy, and according to the sex distribution, so as to calculate the odds ratio of death and to construct the Kaplan-Meier survival curves (Fig. 2). Among 500 patients, 214 patients with diabetes had a significantly higher mortality rate (27.6%) than those without diabetes (17.8%) (*p* < 0.01), with an OR of 1.75. Among patients who received intensive care treatment (*n* = 27), the mortality rate was 66.7%, much higher than that of those who did not need to receive intensive care (19.4%) (*p* < 0.001), and the OR was 8.28. In patients who needed mechanical ventilation treatment (*n* = 48), the mortality rate was 52.1%, much higher than that in patients without mechanical ventilation treatment (18.1%) (*p* < 0.001), and the OR was 6.32. The mortality rate of patients who received steroids was 25.5%, which was higher than the one in those without use of steriods (18.5%) (*p* = 0.058), with an OR of 1.51.

### Characteristics and outcomes of COVID-19 patients with diabetes and without diabetes

We compared the outcomes between diabetic group and non-diabetic group, and found that the mortality rate in patients with diabetes was higher than that in non-diabetic subjects. The mortality rate in patients with diabetes rose more than the one in non-diabetic group, from at discharge (18.7% *vs*. 12.6%, *p* = 0.06) to 6 months after discharge (27.6% *vs*. 17.8%, *p* < 0.01). Furthermore, we used logistic regression analysis to examine the association between diabetes and mortality. The results showed that the mortality rate of patients with diabetes at discharge (OR: 1.649, 95% CI [1.007–2.699], *p* = 0.047) and 6 months after discharge (OR: 1.754, 95% CI [1.145–2.686], *p* = 0.01) were all significantly higher than that of non-diabetic patients. Compared with non-diabetic COVID-19 patients, COVID-19 patients with diabetes had significantly higher infectious and inflammatory indicators (all *p* < 0.05), with procalcitonin increased by 0.077 ng/ml, CRP increased by 26.3 mg/L, WBC increased by 2.23 × 10^9^/L, and neutrophils increased by 2.15 × 10^9^/L. Lymphocyte counts in patients with diabetes decreased by 0.14 × 10^9^/L (*p* < 0.01). In addition, we found that pH and standard bicarbonate in patients with diabetes decreased (*p* < 0.05), while anion gap and blood potassium increased (*p* < 0.01), which indicated exaggerated acidosis in patients with diabetes. There was also a significant increase in levels of myocardial injury markers in the diabetic group (*p* < 0.05), including NT-proBNP, CK and CK-MB, which suggests that patients with COVID-19 infection complicated with diabetes were more likely to have myocardial injury. Notably, the hospitalization expenses of patients with diabetes were markedly higher than those of non-diabetic patients (*p* < 0.05), increased by about 420 US dollars. Furthermore, BMI, systolic blood pressure, osmotic pressure, serum creatinine and D-dimer were significantly different between COVID-19 patients with diabetes and without diabetes (*p* < 0.05). There was no significant difference in age, sex distribution, duration of symptoms before admission, diastolic blood pressure, heart rate, blood sodium, platelet count, troponin and length of hospital stay between two groups. No difference was shown in the proportion of patients who received treatment in ICU, use of steroids or mechanical ventilation. Detailed information for characteristics and outcomes of COVID-19 patients with diabetes and without diabetes was shown in Table 2.

**Table 2:** Characteristics and outcomes of COVID-19 patients with diabetes and without diabetes.

Characteristics	Total (*n* = 500)	Diabetic group (*n* = 214)	Non-diabetic group (*n* = 286)	*p*-value
Demographic characteristics				
Age (year)	74 (60, 84)	75 (62, 84)	72 (58, 84)	0.133
Gender				0.362
Male (*n*)	349 (69.8%)	154 (44.1%)	195 (55.9%)	
Female (*n*)	151 (30.2%)	60 (39.7%)	91 (60.3%)	
BMI (kg/m^2^)	23.03 (20.26, 25.32)	23.7 (19.9, 27.5)	22.8 (20.0, 24.7)	0.004
Clinical characteristics				
Duration of symptoms prior to admission (day)	7 (5, 13)	7 (4, 14)	8 (5, 12)	0.213
Systolic blood pressure (mmHg)	131 (120, 144)	134 (121, 148)	131 (113, 149)	0.018
Diastolic blood pressure (mmHg)	78 (70, 86)	80 (70, 87)	78 (71, 85)	0.328
Heart rate (beat/min)	85 (77, 97)	86 (76, 98)	85 (78, 96)	0.678
HbA1c (%)	6.60 (6.00, 7.50)	7.3 (6.8, 8.9)	5.9 (5.7, 6.2)	0.000
Random plasma glucose (mmol/L)	7.79 (6.10, 11.27)	11.33 (8.06, 16.33)	6.73 (5.82, 7.92)	0.000
Fasting plasma glucose (mmol/L)	6.27 (5.11, 8.73)	8.85 (6.35, 12.25)	5.35 (4.83, 6.57)	0.000
CRP (mg/L)	54.7 (12.7, 129.7)	68.0 (15.3, 142.8)	41.7 (9.2, 117.3)	0.022
pH	7.41 (7.36, 7.45)	7.40 (7.35, 7.45)	7.41 (7.37, 7.45)	0.038
HCO_3_^−^ (mmol/L)	23.30 (19.20, 25.50)	22.1 (18.5, 25.1)	23.7 (20.6, 25.8)	0.001
Anion gap (mmol/L)	11.20 (9.40, 13.60)	11.9 (9.8, 15.3)	10.6 (9.2, 12.6)	0.000
Osmotic pressure (/kg(water))	282 (276, 292)	286 (279, 307)	280 (274, 286)	0.000
Na^+^ (mmol/L)	138 (135, 141)	137.7 (134.4, 141.7)	138.3 (135.0, 141.0)	0.503
K^+^ (mmol/L)	3.87 (3.52, 4.33)	4.0 (3.6, 4.5)	3.8 (3.5, 4.2)	0.001
Serum creatinine (μmoI/L)	91 (70, 151)	107 (77, 179)	82 (66, 127)	0.000
D-dimer (mg/L)	0.94 (0.45, 2.04)	1.22 (0.62, 2.54)	0.73 (0.38, 1.66)	0.000
Procalcitonin (ng/ml)	0.13 (0.06, 0.50)	0.19 (0.07, 0.70)	0.11 (0.05, 0.32)	0.000
White blood cell count (10^9^/L)	7.33 (4.97, 10.16)	8.6 (6.4, 12.1)	6.4 (4.6, 8.8)	0.000
Neutrophil (10^9^/L)	5.56 (3.40, 8.59)	6.9 (4.6, 10.5)	4.7 (3.0, 7.0)	0.000
Lymphocyte (10^9^/L)	0.82 (0.53, 1.26)	0.76 (0.50, 1.15)	0.90 (0.60, 1.35)	0.005
Platelet count (10^9^/L)	202 (152, 272)	213 (152, 277)	196 (154, 269)	0.511
Troponin (μg/L)	0.07 (0.01, 7.15)	0.11 (0.02, 9.45)	0.06 (0.01, 6.00)	0.053
NT-proBNP (ng/L)	164 (50, 795)	215 (71, 1130)	124 (32, 627)	0.002
Creatine kinase (U/L)	94 (52, 227)	132 (55, 300)	83 (49, 182)	0.007
Creatine phosphokinase isoenzyme (U/L)	14 (10, 19)	16 (11, 23)	13 (9, 17)	0.000
Length of stay in hospital (day)	9 (7, 15)	10 (7, 16)	9 (7, 14)	0.263
Hospitalization expenses ($)	2,088 (1,312, 3,605)	2,355 (1,483, 4,177)	1,933 (1,258, 3,285)	0.019
Therapy				
Treatment in ICU (*n*)	27 (5.4%)	11 (40.7%)	16 (59.3%)	0.824
Use of steriods (*n*)	251 (50.2%)	111 (44.2%)	140 (55.8%)	0.518
Mechanical ventilation (*n*)	48 (9.6%)	18 (37.5%)	30 (62.5%)	0.435

**Note:**

Data were presented as *n* (%) or mean ± SD or median (range) unless otherwise stated. Abbreviations: BMI, body mass index; CRP, C-reactive protein.

### Characteristics and outcomes of survivors and non-survivors of COVID-19 patients with diabetes

We further made comparisons between the survivors and non-survivors of 214 COVID-19 patients with diabetes. Compared with survivors, non-survivors were older in age (*p* < 0.001) with higher heart rate (*p* < 0.005). The proportion of female was higher in non-survivors (*p* < 0.05). There was no significant difference in BMI, the duration of symptoms before admission, blood pressure, HbA_1c_, lymphocyte, platelet, troponin and hospitalization expenses between two groups. The acidosis, infection and inflammation seemed to be more serious in non-survivors. with bicarbonate decreased by 3.76 mmol/L (*p* < 0.01), pH decreased by 0.03 (*p* = 0.01), anion gap increased by 3.05 mmol/L (*p* < 0.001), blood potassium increased by 0.21 mmol/L (*p* = 0.025), CRP increased by 70.6 mg/L (*p* < 0.01), procalcitonin increased by 0.42 ng/ml (*p* < 0.001), WBC and neutrophils respectively increased by 4.26 × 10^9^/L and 3.18 × 10^9^/L (*p* < 0.01). Besides, there was also a notable increase in markers of myocardial injury (NT-proBNP, CK, CK-MB) in non-survivors (*p* < 0.05). The serum creatinine of non-survivors was significantly increased by 80 μmoI/L (*p* < 0.01), which indicates that renal insufficiency was more common in non-survivors. Compared with survivors, non-survivors had worse blood glucose control, with random blood glucose increased by 3.60 mmol/L (*p* < 0.05), and fasting blood glucose increased by 2.65 mmol/L (*p* < 0.01). The osmotic pressure of survivors was lower than the ones of non-survivors (*p* < 0.01). Besides, we found that the mortality rate in patients who received intensive care treatment (72.7%) and patients who needed mechanical ventilation treatment (66.7%) were much higher than that in patients without intensive care treatment (27.3%) or mechanical ventilation treatment (33.3%) (*p* < 0.01). Table 3 showed the characteristics and outcomes of survivors and non-survivors of COVID-19 patients with diabetes.

**Table 3:** Characteristics and outcomes of survivors and non-survivors of COVID-19 patients with diabetes.

Characteristics	Total (*n* = 214)	Survivor (*n* = 155)	Non-survivor (*n* = 59)	*p*-value
Demographic characteristics				
Age (year)	75 (62, 84)	71 (59, 81)	83 (75, 88)	0.000
Gender				0.028
Male (*n*)	154 (72.0%)	118 (76.6%)	36 (23.4%)	
Female (*n*)	60 (28.0%)	37 (61.7%)	23 (38.3%)	
BMI (kg/m^2^)	23.74 (19.94, 27.54)	23.87 (20.09, 27.65)	23.12 (19.20, 27.05)	0.501
Clinical characteristics				
Duration of symptoms prior to admission (day)	7 (4, 13)	10 (8, 16)	9 (4, 16)	0.327
Systolic blood pressure (mmHg)	134 (121, 148)	134 (120, 146)	136(114, 158)	0.886
Diastolic blood pressure (mmHg)	80 (70, 87)	80 (70, 87)	78 (66, 90)	0.454
Heart Rate (beat/min)	86 (76, 98)	84 (75, 95)	92 (75, 109)	0.002
HbA_1c_ (%)	7.3 (6.8, 8.9)	7.3 (6.8, 8.4)	7.6 (6.8, 10.0)	0.190
Random plasma glucose (mmol/L)	11.33 (8.06, 16.33)	10.88 (7.88, 14.87)	14.48 (7.68, 21.28)	0.017
Fasting plasma glucose (mmol/L)	8.85 (6.35, 12.25)	8.27 (6.22, 10.92)	10.92 (7.36, 14.31)	0.003
Diabetic complications (*n*)	81 (37.9%)	44 (54.3%)	37 (45.7%)	0.000
Diabetic ketoacidosis (*n*)	28 (13.1%)	12 (42.9%)	16 (57.1%)	0.000
Diabetic nephropathy (*n*)	56 (26.2%)	32 (57.1%)	24 (42.9%)	0.003
Hyperglycemic hyperosmolar coma (*n*)	8 (3.7%)	3 (37.5%)	5 (62.5%)	0.064
CRP (mg/L)	68.0 (15.3, 142.8)	51.7 (14.4, 113.8)	122.3 (15.3, 187.2)	0.001
pH	7.40 (7.35, 7.45)	7.41 (7.36, 7.45)	7.38 (7.32, 7.44)	0.010
HCO_3_^−^ (mmol/L)	21.58 (17.02, 26.14)	23.10 (19.05, 25.65)	19.34 (14.64, 24.04)	0.000
Anion Gap (mmol/L)	11.90 (9.80, 15.28)	11.20 (9.65, 14.25)	14.25 (11.00, 16.70)	0.001
Osmotic pressure (/kg(water))	286 (279, 307)	283 (277, 292)	309 (289, 321)	0.000
Na^+^ (mmol/L)	137.7 (134.4, 141.7)	137.0 (134.1, 140.4)	139.1 (135.6, 147.2)	0.000
K^+^ (mmol/L)	4.00 (3.58, 4.45)	3.99 (3.56, 4.43)	4.20 (3.68, 4.73)	0.025
Serum creatinine (μmol/L)	107 (77, 180)	94 (75, 139)	174 (98, 304)	0.000
D-dimer (mg/L)	1.22 (0.62, 2.54)	0.95 (0.47, 1.65)	2.76 (1.36, 9.36)	0.000
Procalcitonin (ng/ml)	0.19 (0.07, 0.70)	0.14 (0.06, 0.55)	0.56 (0.21, 3.53)	0.000
White blood cell count (10^9^/L)	8.59 (6.40, 12.14)	7.59 (6.12, 10.66)	11.85 (6.07, 17.63)	0.000
Neutrophil (10^9^/L)	6.87 (4.55, 10.48)	6.30 (4.32, 9.27)	9.48 (4.46, 14.50)	0.000
Lymphocyte (10^9^/L)	0.76 (0.50, 1.15)	0.77 (0.52, 1.22)	0.68 (0.45, 1.00)	0.221
Platelet count (10^9^/L)	213 (152, 277)	216 (143, 280)	200 (154, 258)	0.497
Troponin (μg/L)	0.11 (0.02, 9.45)	0.06 (0.01, 10.30)	0.15 (0.05, 45.55)	0.188
NT-proBNP (ng/L)	215 (71, 1,130)	145 (54, 493)	1,090 (244, 3,215)	0.000
Creatine Kinase (U/L)	132 (55, 300)	102 (53, 246)	247 (73, 379)	0.002
Creatine phosphokinase isoenzyme (U/L)	16 (11, 23)	15 (11, 22)	17 (13, 27)	0.027
Length of stay in hospital (day)	10 (7, 16)	10 (8, 16)	9 (4, 16)	0.018
Hospitalization expenses ($)	2,356 (1,484, 4,179)	2,266 (1,521, 3,994)	2,713 (1,446, 4,908)	0.377
Therapy				
Treatment in ICU (*n*)	11 (5.1%)	3 (27.3%)	8 (72.7%)	0.002
Use of steriods (*n*)	111 (51.9%)	82 (73.9%)	29 (26.1%)	0.624
Mechanical ventilation (*n*)	18 (8.4%)	6 (33.3%)	12 (66.7%)	0.000

**Note:**

Data were presented as n (%) or mean ± SD or median (range) unless otherwise stated. Abbreviations: BMI, body mass index; CRP, C-reactive protein.

**Figure 3:**
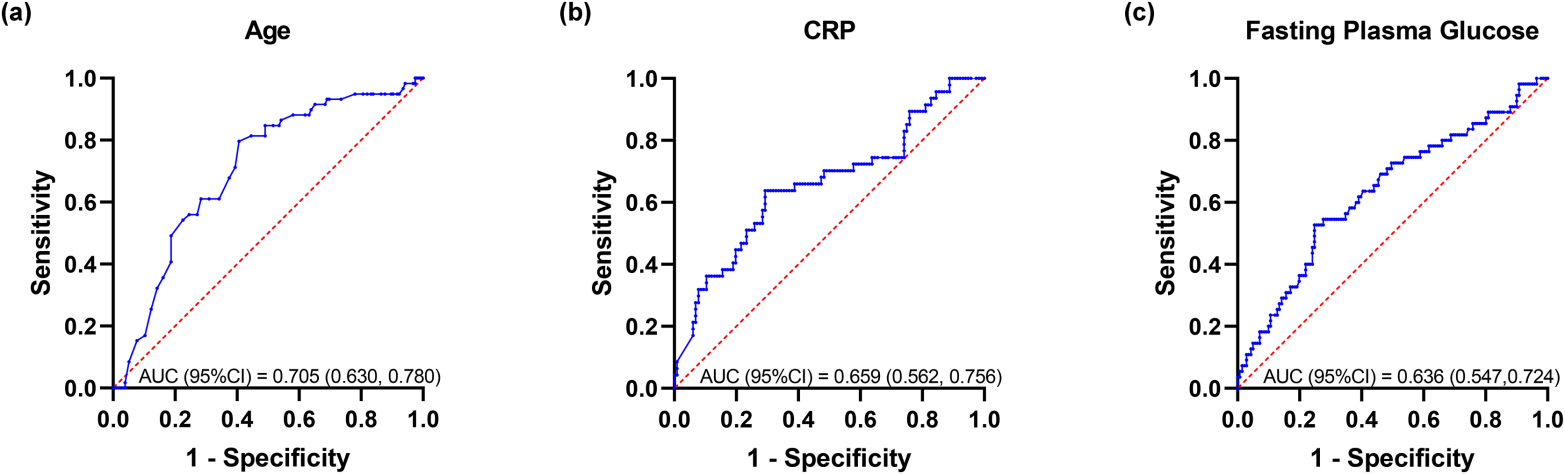
ROC curves of patients with diabetes after COVID-19 infection. (A) Age; (B) CRP; (C) Fasting blood glucose. Abbreviations: CRP, C-reactive protein.

**Figure 4:**
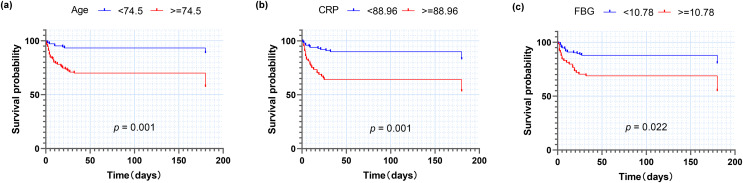
Kaplan-Meier analysis for death of patients with diabetes after COVID-19 infection. (A) Age; (B) CRP; (C) Fasting blood glucose. Abbreviations: CRP, C-reactive protein.

Based on univariate analysis, multivariate logistic regression analysis was performed to further determine the factors related to death (Table 4). The results showed that among COVID-19 patients with diabetes, those with older age and higher CRP had significantly higher mortality (adjusted *p*-value < 0.05), with trends noted for those with higher higher fasting blood glucose (adjusted *p*-value < 0.1). There was no correlation between death and heart rate, serum creatinine, anion gap or platelet count. ROC curve analysis was conducted to investigate the associations between the following variables and death (Fig. 3), including age, CRP and fasting blood glucose. Chi-squared test was used to calculate the corresponding odds ratio for predicting death. Among 214 COVID-19 patients with diabetes, the age of 74.5 years old is the optimal cut-off for predicting death, with sensitivity of 79.7%, specificity of 59.4%, the area under ROC curve (AUC) of 0.705 and OR of 5.72. Similarly, we calculated CRP (optimal cut-off = 88.96 mg/L, sensitivity = 63.8%, specificity = 70.7%, AUC = 0.659, OR = 4.26) and fasting blood glucose (critical value = 10.78 mmol/L, sensitivity = 52.7%, specificity = 75.2%, AUC = 0.636, OR = 3.38), Moreover, the Kaplan-Meier survival curves with the above-mentioned optimal critical values were constructed (Fig. 4).

**Table 4:** Multivariate logistics regression analysis of the death of COVID-19 patients with diabetes risk factors.

Covariate	OR	95% CI	*p*-value	Adjusted *p*-value
Age	1.101	[1.028–1.178]	0.006	0.027
Heart rate	0.997	[0.956–1.039]	0.884	0.884
Fasting plasma glucose	1.129	[1.011–1.261]	0.032	0.096
CRP	1.016	[1.005–1.026]	0.003	0.027
Serum creatinine	1.002	[0.996–1.007]	0.546	0.614
D-dimer	1.047	[0.993–1.103]	0.087	0.157
Anion gap	0.933	[0.798–1.090]	0.380	0.57
Platelet count	1.002	[0.997–1.007]	0.437	0.562
Creatine phosphokinase isoenzyme	1.045	[0.996–1.096]	0.074	0.167

**Note:**

Abbreviations: CRP, C-reactive protein.

In addition, the mortality rate of patients with diabetic complications (45.7%) was significantly higher than that of those without diabetic complications (19.8%) (*p* < 0.001), and the OR value was 4.24. In our study, diabetic complications mainly included diabetic ketoacidosis (*p* < 0.001, OR = 4.43), diabetic kidney disease (*p* < 0.01, OR = 2.64) and hyperglycemic hyperosmolar coma (*p* = 0.064, OR = 4.69).

## Discussion

In this study, we investigated the survival of Chinese hospitalized patients with COVID-19 infection during a specific outbreak. The mortality rate of COVID-19 patients with diabetes was found to be 1.5 times higher than that of patients without diabetes, along with higher occurrence of other adverse clinical and socioeconomic outcomes. We suggest that this may be related to the degree of inflammation (leukocytes, granulocytes, CRP, procalcitonin). The blood glucose control indicators (random blood glucose, fasting blood glucose) has a trend towards significance. These results emphasize the importance of controlling inflammation and blood sugar in managing COVID-19 infection in patients with diabetes.

As mentioned above, we found that COVID-19 patients with diabetes had more severe inflammation with significantly higher WBCs, neutrophils, CRP, and procalcitonin than those without diabetes. Many previous studies have also shown that diabetes correlates with the incidence and severity of lung diseases (Ehrlich et al., 2010; Equils et al., 2016). Individuals with diabetes are more vulnerable to primary or secondary infection of pathogenic microorganisms, and post-infection conditions are worse (Nabi, Ebihara & Shekhar, 2023). According to Ehrlich et al. (2010), the incidence of asthma, chronic obstructive pulmonary disease, pulmonary fibrosis and pneumonia in diabetic patients is higher than that in non-diabetic patients, and with the increase of HbA_1c_ in patients with diabetes, the risk of COPD and pneumonia increases significantly (Ehrlich et al., 2010). The mechanical effects of hyperglycemia on inflammation and cytokines surge have been confirmed in many studies (Mirzaei et al., 2021; Vasbinder et al., 2022). Hyperglycemia can cause damage to phagocytosis and bactericidal activity, neutrophil chemotaxis, complement fixation and microbial conditioning, as well as changes in chemokines (Berbudi et al., 2020). In addition, hyperglycemia and enhanced glycolysis can activate HIF-1α and the production of mitochondrial reactive oxygen species to increase the replication of novel coronavirus in human monocytes (Codo et al., 2020). In the case of coronavirus infection, excessive immune and stress response will lead to the release of inflammatory cytokines, exacerbating the severity of the disease, which is also one of the reasons for the high mortality of COVID-19 patients with diabetes. However, lymphocytes, as one of the inflammatory indicators, were significantly lower in COVID-19 patients with diabetes than in those without diabetes. This is because the targeted infection of COVID-19 particles enable to destroy the cytoplasmic components of lymphocytes and lead to their destruction, making lymphopenia as a common feature of COVID-19 infected subjects (Yang et al., 2020). Significantly lower lymphocyte counts were also observed in patients with diabetes in our study.

Acute respiratory distress syndrome (ARDS) is one of the main manifestations of severe COVID-19 infection, and is also one of the main causes of acidosis in patients. Some studies have found that in patients with diabetes, deoxyhemoglobin is higher than that in nondiabetic individuals, and deoxyhemoglobin may be more vulnerable to be attacked by the surface protein of the new coronavirus, further aggravating the degree of respiratory distress (Bielka, Przezak & Pawlik, 2021). In addition, the effect of diabetic microvascular disease on the lung is often ignored. Abnormal small blood vessels and thickening of capillary basement membrane in the lungs of patients with diabetes usually lead to subclinical pulmonary dysfunction, thereafter reduce lung capacity and diffusion ability (Chance et al., 2008). In a cross-sectional study, non-smoking T2DM patients had significantly decreased peak oxygen uptake, pulmonary blood flow, pulmonary diffusion capacity and capillary blood volume during exercise, and this dysfunction was associated with blood glucose control status measured by HbA_1c_ level (Chance et al., 2008; Mazucanti & Egan, 2020). In our study, we also found that COVID-19 patients with poor glycemic control had a higher mortality rate, measured by random plasma glucose and fasting plasma glucose.

Except invading lung surface cells and causing pneumonia, COVID-19 can cause damage to cardiomyocytes (Oudit et al., 2009; Li et al., 2020a). Oudit et al. (2009) found that during the outbreak of COVID-19 in Toronto, COVID-19 virus was detected in heart samples of 35% of those who died of COVID-19 infection (Zhang, Li & Niu, 2020). They also confirmed that the lung infection of COVID-19 in mice can lead to myocardial infection through ACE2, which is a crucial part of renin-angiotensin system and is widely distributed in the heart, kidneys, and lungs (Oudit et al., 2009; Zhang, Li & Niu, 2020). Moreover, some studies have demonstrated that diabetes can increase the expression of ACE2 by activating the LTB4 pathway under COVID-19 infection, thus increasing the degree of myocardial injury (Bonyek-Silva et al., 2021). Hypoxemia caused by COVID-19 could also be an important cause of heart injury. Pneumonia can cause significant gas exchange disorder and hypoxemia, reduce the energy supply of cell metabolism, increase anaerobic fermentation, cause intracellular acidosis and oxygen free radicals to destroy the phospholipid layer of cell membrane. Hypoxia induced calcium influx can also lead to myocardial cell damage and apoptosis (Li et al., 2020a). In this study, COVID-19 patients with diabetes also demonstrated higher levels of myocardial injury indicators in univariate analysis.

Some studies have found virus-like particles in podocytes and renal tubular epithelial cells, and determined that the functional receptor ACE2 of the new coronavirus was highly expressed in the kidney, which indicates that there may be a direct cytopathic effect of the virus on the kidney, leading to renal damage in patients with infection (Perrotta et al., 2023). In addition to the direct infection of virus on renal cells, the inflammatory reaction caused by cytokine storm in COVID-19 patients can also result in renal tissue damage (Zhang et al., 2020; Diao et al., 2021). Inflammation and endothelial injury further increase procoagulant factors, cause intravascular injury, and may eventually lead to thrombosis and ischemia, thus promote the development of kidney injury (Milbrandt et al., 2009; Zhou et al., 2020). In our study, we also found that the serum creatinine of 10.8% of COVID-19 patients without a history of kidney disease exceeded the upper limit (133 μmol/L), and the serum creatinine and D-dimer levels of patients with diabetes were significantly higher than those of non-diabetic patients in univariate analysis. Although serum creatinine and D-dimer levels were not significant in multivariate analysis, diabetes is an independent risk factor for the progression of acute kidney injury (AKI), and hyperglycemia can aggravate AKI induced by renal ischemia-reperfusion injury/hypoxia reperfusion injury (Kale et al., 2022). In addition, insulin resistance and hyperglycemia can also lead to vascular endothelial dysfunction, coagulation fibrinolysis imbalance and high reactivity of platelets, thus lead to easier thrombosis in diabetic patients (Ye et al., 2020).

In the present study, among COVID-19 patients with diabetes, non-survivors often have poor blood glucose control, which is consistent with the previous studies. Some studies have shown that the mortality rate of patients with blood glucose ranging from 3.9 to 10.0 mmol/L is lower than that of patients with blood glucose level higher than 10.0 mmol/L (Bode et al., 2020). With the increase of glucose level, the infection rate and replication rate of the virus increase, which suggests that patients with hyperglycemia may have a higher risk of COVID-19 infection in different organs and experience more serious conditions after infection (Memon & Abdelalim, 2021). In addition, compared with patients with high blood glucose levels, those with satisfied blood glucose control have less incidence of pneumonia, lower levels of neutrophil count, CRP, interleukin-6 and procalcitonin, which is also in accordance with the results of our study and the above-mentioned mechanism (Drucker, 2021). As reported by Mazucanti & Egan (2020) blood glucose control affects the pulmonary function of patients with diabetes (Chance et al., 2008). Furthermore, diabetes can cause ketoacidosis, hyperglycemic hyperosmolar coma, nephropathy and other serious diseases, thus increase the risk of death and reduce the overall quality of life (Zhang et al., 2022). Therefore, better glycemic management and prevention of diabetic complications can improve the prognosis of COVID-19 patients and reduce their mortality.

Regarding the relationship between diabetes and COVID-19, we should also pay attention to the fact that COVID-19 infection may cause hyperglycemia and diabetes. Several studies have reported new-onset diabetes associated with the presence of COVID-19. A study of hospitalized patients from Wuhan showed that 21% of them were newly diagnosed with diabetes and 28.4% were diagnosed with dysglycemia (Li et al., 2020b). Sathish et al. (2021) demonstrated that the prevalence rate of new-onset diabetes was 14.4% among 711 patients in COVID-19. However, the exact mechanism behind new-onset hyperglycemia and diabetes in COVID-19 patients is still unclear, which may be related to complicated factors, such as stress hyperglycemia, pre-hospital diabetes and steroid-induced diabetes (Khunti et al., 2021). In our study, it is also difficult to confirm the time sequence of hyperglycemia and COVID-19 infection in patients with hyperglycemia or newly-diagnosed diabetes who were first found at the time of admission, which made it hard to determine the cause-and-effect relationship. But we tried to preclude the conditions of short-term hyperglycemia by chosing HbA_1c_ as the determinant of diabetes.

As far as gender differences are concerned in COVID-19 infection, the relevant research is still relatively lacking, and so far it has not been clear about its impact on mortality. According to a study in New York, the prevalence and the mortality associated with COVID-19 was higher in males than in females (Chen et al., 2020). Another gender analysis of hospitalized COVID-19 patients in New Orleans showed that rate of hospitalization, admission to ICU and in-hospital death was similar in women and men (Yoshida et al., 2021). In our study, we found that the proportion of female was higher in non-survivors, but this may be because the average age of female patients was older than that of male patients.

Concerning socioeconomic outcomes, Alkundi et al. (2020) found that COVID-19 patients with diabetes had longer hospital stay length than patients without diabetes. Although we observed that length of hospital stay were similar between COVID-19 patients with diabetes and without diabetes, we found that diabetes apparently raised the medical expenses of COVID-19 patients. A study carried out in Europe demonstrated that during the first wave of COVID-19, the medical expenses of COVID-19 patients with diabetes far exceeded those of COVID-19 patients without diabetes (Bain et al., 2021). We reasonably speculate that this is due to the higher risk of COVID-19-related serious consequences in patients with diabetes, such as more serious acute respiratory distress, myocardial damage and kidney injury.

The effects of COVID-19 infection could be long-lasting. It is well-recognized that diabetes is a key risk factor for poor prognosis in COVID-19 infection (Mantovani et al., 2020). After 6 months’ follow-up, we found that the mortality rate of patients with diabetes was higher than that of non-diabetic patients. Many studies have demonstrated similar results. During the 10-month follow-up study, it was found that patients were more prone to stroke and cardiovascular diseases than the control group (Khamidullina et al., 2024). Kingery et al. (2021) observed that the readmission rate of patients with diabetes was higher than that of non-diabetic patients during the 30-day follow-up period after recovered from COVID-19 infection and discharged from hospital. Furthermore, the risk of severe COVID-19 reinfection was reported to be higher among patients with diabetes, which would lead to higher mortality (Murillo-Zamora et al., 2021).

In summary, the present study investigated the characteristics, outcomes and associated risk factors in Chinese patients with COVID-19 infections, especially in COVID-19 patients with diabetes, under special context. We believe that our results provide valuable supplements for a better understanding of COVID-19. But we have to admit that our study has some limitations. First, our study was conducted in a single center during a specific epidemic and included hospitalized patients. Second, the individuals were generally old and were complicated with chronic diseases, which may contribute to a higher mortality compared to other studies. Therefore, the results should be interpreted with caution.

## Conclusions

Compared with non-diabetic patients, individuals with diabetes infected with COVID-19 had significantly higher mortality rate and more adverse outcomes, which were related to diabetes control indicators.

## Supplemental Information

10.7717/peerj.18865/supp-1 Supplemental Information 1 Raw data.

10.7717/peerj.18865/supp-2 Supplemental Information 2 STROBE Statement.

10.7717/peerj.18865/supp-3 Supplemental Information 3 Codebook to convert numbers to their respective factors.

## Additional Information and Declarations

### Data Availability

The following information was supplied regarding data availability:

The raw data are available in the Supplemental File.
