# Supplementary material for: Correlation of diabetes and adverse outcomes in hospitalized COVID-19 patients admitted to a tertiary hospital in China during a small-scale COVID-19 outbreak
Source: PeerJ. 2025 Jan 27;13:e18865. doi: 10.7717/peerj.18865 (PMC11781264; doi:10.7717/peerj.18865)
Supplement: Supplemental Information 2 [file peerj-13-18865-s002.doc]

STROBE Statement—Checklist of items of this article.

|  | Item No | Recommendation |
| --- | --- | --- |
| **Title and abstract**  [Lines 1-4 and 17-39] | 1 | (*a*) Indicate the study’s design with a commonly used term in the title or the abstract. |
|  |  | (*b*) Provide in the abstract an informative and balanced summary of what was done and what was found. |
| Introduction | | |
| Background/rationale  [Lines 41-62] | 2 | Explain the scientific background and rationale for the investigation being reported. |
| Objectives  [Lines 63-72] | 3 | State specific objectives. |
| Methods | | |
| Setting  [Lines 76-78] | 4 | Describe the setting, locations, and relevant dates, including periods of recruitment, follow-up, and data collection. |
| Participants  [Lines 75-86] | 5 | Give the eligibility criteria, and the sources and methods of selection of participants. Describe methods of follow-up. |
| Variables  [Lines 87-99] | 6 | Describe the collected variables. |
| Quantitative variables  [Lines 101-105] | 7 | Explain how quantitative variables were handled in the analyses. |
| Statistical methods  [Lines 100-109] | 8 | (*a*) Describe all statistical methods. |
|  |  | (*b*) Describe all drawing tools. |
| Results | | |
| Descriptive data  [Lines 113-123, 137-159 and 162-182] | 9* | (a) Give characteristics of study participants (eg demographic, clinical, social). |
|  |  | (b) Indicate number of participants for each variable of interest. |
| Outcome data  [Tables 1, 2 and 3] | 10* | Report numbers of outcome events or summary measures over time. |
| Main results  [Tables 1, 2 and 3] | 11 | (*a*) Give variables estimates and their precision (eg, 95% confidence interval). |
|  |  | (*b*) Report category boundaries when continuous variables were categorized. |
| Other analyses  [Lines 100-109] | 12 | Report other analyses done—eg ROC curve analysis, and odds ratio. |
| Discussion and conclusion | | |
| Key results  [Lines 207-311] | 13 | Summarise key results with reference to study objectives. |
| Limitations  [Lines 312-315] | 14 | Discuss limitations of the study, taking into account sources of potential bias or imprecision. |
| Interpretation  [Lines 318-320] | 15 | Give a cautious overall interpretation of results considering objectives, limitations, multiplicity of analyses, results from similar studies, and other relevant evidence. |
| Other information | | |
| Funding  [Lines 326-329] | 16 | Give the source of funding and the role of the funders for the present study. |

*Give information separately for different groups.
